# Optimizing nutrient removal and biomass production of the Algal Turf Scrubber (ATS) under variable cultivation conditions by using Response Surface Methodology

**DOI:** 10.3389/fbioe.2022.962719

**Published:** 2022-09-06

**Authors:** Xinyu Gan, Holger Klose, Diana Reinecke

**Affiliations:** ^1^ IBG2-Plant Sciences, Forschungszentrum Jülich GmbH, Jülich, Germany; ^2^ Faculty of Agriculture, University of Bonn, Bonn, Germany; ^3^ RWTH Aachen University, Aachen, Germany

**Keywords:** algal turf scrubber, wastewater treatment, bioremediation, phosphorous removal, algal biomass

## Abstract

This study investigated and optimized the nutrient remediation efficiency of a simple low-cost algal biofilm reactor, the algal turf scrubber (ATS), for wastewater treatment. Combined effects of three cultivation variables—total inorganic carbon, nitrogen-to-phosphorous (N:P) ratio, and light intensity—were examined. The ATS nutrient removal efficiency and biomass productivity were analyzed considering the response surface methodology (RSM). The maximum removal rates of total P and N were 8.3 and 19.1 mg L^−1^ d^−1^, respectively. As much as 99% of total P and 100% of total N were removed within 7 days. Over the same period, the dissolved oxygen concentration and pH value of the medium increased. The optimal growth conditions for simultaneous maximum P and N removal and biomass productivity were identified. Our RSM-based optimization results provide new insights into the combined effect of nutrient and light availability on the ATS remediation efficiency and biomass productivity.

## Graphical Abstract

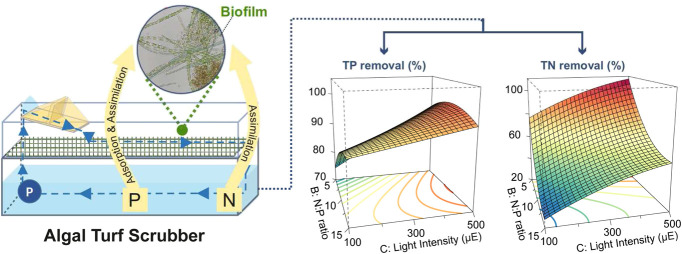

## 1 Introduction

The vast quantity of nutrient-rich, urban, agricultural, and industrial wastewater (WW) generated by an ever-increasing human population and its activities poses a threat to natural bodies of water (Pittman et al., 2011; Daud et al., 2015). Eutrophication is one of the most striking effects of nutrient release, and it is associated with the development of harmful algal blooms and anoxic zones (Qin, 2009; Dodds and Smith, 2016). Furthermore, nitrogen leakage into drinking water can negatively affect human health (Wegahita et al., 2020), while mining and the depletion of finite phosphorous ores can cause complex environmental and political issues (Barquet et al., 2020). Therefore, WW treatment and nutrient recovery are essential for healthy ecosystems and human populations.

**FIGURE 1:**
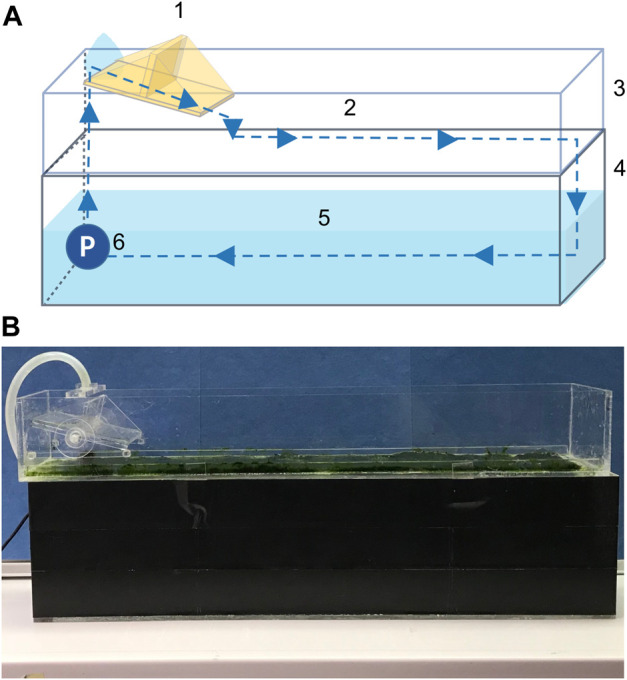
Schematic drawing **(A)** and photograph **(B)** of the lab-scale algal turf scrubber (ATS). Components are (1) tipping bucket, (2) mesh for biofilm attachment, (3) flow-way, (4) container, (5) medium, and (6) pump. Note the container was covered with opaque material to limit the light influx.

Among the numerous physical and chemical WW treatment methods, biological remediation technologies based on algae have been particularly attractive due to their high nutrient removal efficiency and comparatively low land requirements (Wang et al., 2018; Li et al., 2019). Furthermore, algal biomass can serve as an intermediate nutrient carrier between the WW and crop production. However, harvesting algal biomass of suspended cultures, such as open ponds and tubular photobioreactors, is time- and labor-intensive (Richardson et al., 2012). Harvesting processes can account for as much as 20–30% of the total production costs in suspended culture systems (Demirbas, 2010). In contrast, algal biofilm reactors are more cost-effective due to the higher biomass density and easier down-stream processing. In algal biofilm reactors, the biofilm constitutes an environmental mesocosm of bacteria, pro- and eukaryotic algae, fungi, and protozoa attached to a matrix and submerged in WW. The biofilm is harvested by scraping off the biomass. This process is illustrated in Figure 1 (Christenson and Sims, 2011). One of these systems, the algal turf scrubber (ATS), has been successfully employed in the treatment of manure, agricultural drainage, and urban wastewater (Mulbry et al., 2008; Walter et al., 2008; Higgins and Kendall, 2011; D’Aiuto et al., 2015; Ray et al., 2015). The system offers ecological benefits such water purification and oxygenation, as well as CO_2_ fixation by the algae. The ATS biomass can be used either as a long-term fertilizer and soil conditioner, or as animal feed (carbohydrates, proteins, and lipids) (Ray et al., 2015; Schreiber et al., 2018; Marella et al., 2019; Gray, 2021).

In ATS biofilms, the various algal species use a broad range of uptake and turn-over mechanisms to meet their macro- and micronutrient requirements. In addition, essential macronutrients, such as nitrogen and phosphorous, can be precipitated and assimilated as reserves. Depending on the nutrient quantity and quality and the algal species, the major enzymes in the nitrogen fixation are the glutamate dehydrogenase, glutamate ammonia ligase, and glutamine oxoglutarate aminotransferase. The main group of enzymes in the phosphorous recovery are the alkaline phosphatases and Pi uptake transporters at pH 9–11 (Nurdogan and Oswald, 1995; Sañudo-Wilhelmy et al., 2004; Su et al., 2011; Xu et al., 2014). Furthermore, the efficiency of nutrient removal by algal cultures, including ATS biofilms, is strongly affected by the culture conditions. These conditions include osmolarity, shear force, retention time, temperature, light quantity and quality, as well as biotic factors (D’Aiuto et al., 2015; Liu et al., 2016). To improve the efficiency and economics of nutrient removal in ATS systems, it is critical to investigate the relationship between these culture conditions and ATS performance. Although ATS systems have been used in WW treatment for 40 years, there remains a need for further systematic studies to optimize the ATS system, Table 1.

**TABLE 1:** The settings and performances of reviewed algal turf scrubber (ATS) systems.

ATS System			Wastewater			Cultivation Conditions		Effluent (mg L^−1^ d^−1^)			Biomass		References
Size (m); Slope (%)	Flow rate (L^−1^); Intervals (min^−1^)	Replicates; Site	Source; volume (L)	Nutrient concentration (mg L^−^)	pH-value	T (°C); Light intensity (µmol photons m^−2^ s^−1^)	Harvest cycle (d)	Removal (mg L^−1^ d^−1^)		pH-value	Biomass productivity (g m^−2^ d^−1^)	Nutrient and ash content (%)	
								P	N				
0.5 × 10; 1%	46.5; zero	Singlicate; outdoor	Reservoir	TP: 0.002–0.108 TN: 1.9–3.3	7.01	15.0–26.9; N/A	3–9	18–49	161–214	7.42	17.6–25.4	P: 0.1–0.2 Ash: 87.2	Chen et al. (2015)
1 × 50; 2%	60–700 L min^−1^ m^−1^; zero	6 flow-ways; outdoor	Agricultural drainage; 1,200	TP: <0.1 TN: <0.5	N/A	N/A; N/A	7	25mg m^−2^ d^−1^ or 50–69%	125 mg m^−2^ d^−1^or 53–72%	N/A	N/A	P: 0.21–0.26 Ash: 60–70	Kangas and Mulbry, (2014)
0.3 × 90; 2%	60; 5–6	Singlicate; outdoor	River	PO_4_-P: 0.03–0.09 NO_3_-N: 0.4–1.4	N/A	5–30; N/A	7–21	3–40 mg m^−2^ d^−1^	30–450	N/A	11–18	P: 0.2 Ash: 60–70	Kangas et al. (2017)
1 × 1; > N/A	110; 4	Singlicate; indoor	Diluted manure effluent; 200	TN: 1.3–9.0	7–7.5	19–24; 240–633	7	0.6–2.4 mg L^−1^ d^−1^ loading	3.8–17.4 mg L^−1^ d^−1^ loading	7–7.5 (CO_2_ controlled)	5–9	P: 0.6–1.5 Ash: 7–10	Kebede-westhead et al. (2003)
0.39 × 2.5;1%	2, 4, 6, 8; N/A	Triplicate; outdoor	Horticultural drainage; 65	PO_4_-P: 9–12; NO_3_-N: 30–50	7.0	N/A; N/A	7	0.6–1.2 99%	1–3 or <99%	>8.5	2.0	P: 2.1–2.3 N: 6.2–6.8	Liu et al. (2016)
0.1 × 0.75; 1%	65; zero	Singlicate with 3 cycles; outdoor	Non-point source WW	TP: 3.7–4.4 TN: 51–69	8–8.8	20–32; 781–1,147	15	0.4–1.25	1.3–2.5	N/A	20.7–38.9	P: 0.9–3.2 N: 5.0–6.4	Marella et al. (2019)
1 × 30; 1 or 2%	93; 4–8	Duplicate; outdoor	Diluted manure effluent;3,500	TP: 0.68–3.6 TN: 2.6–21.4	7.0	<32; N/A	4–12	0.4	2,500	9–10	2.5–24	P: < 1.0 N: 6.8	Mulbry et al. (2008)
3 × 30; 2%	750; 4	Singlicate; outdoor	Stream	TP: 0.25 TN: 4.1	7.8	15–25; N/A	5–14	48%	12%	10.8	12–34	N/A	Sandefur et al. (2011)
0.5 × 1; 0.5%	25;N/A	Triplicate; outdoor	Diluted anaerobically digested food-waste concentrate	TP: 13 TN: 164	7.2	22–28; 6,000–8,000 μmol photons m^−2^	7	0.02–0.18 g m^−2^ d^−1^	0.27–1.65 g m^−2^ d^−1^	9.3–10.1	20–25	P: 0.8–2.1 N: 8.0–9.9	Sutherland et al. (2020)
0.1 × 0.52; 1%	0.3; 8–10	Triplicate; indoor	Artificial WW; 5	TP: 10 TN: 50–150	7–7.2	22–24; 100, 300, 500	7	7.5–10.4 mg L^−1^ or <99%	35.2–64.7 mg L^−1^ 100%	10.5–11.2	4.1–11.2	P: 1.1–1.9 N: 4.9–7.8 Ash: 6.3–9.4	Current study

Note: N/A means nothing was reported in the reference.

Response surface methodology (RSM) is a collection of mathematical and statistical techniques for designing experiments, building models, evaluating the interdependence of variables, and obtaining the optimal response conditions with a limited number of planned experiments. The Box–Behnken design (BBD) (Ferreira et al., 2007) is one of RSMs. In short, the model has three levels for each variable and is built specifically to fit a quadratic model. Compared to the full factorial design, the BBD largely reduces the number of necessary experiments. Furthermore, the RSM has been successfully used to model the growth of microalgae (Shen et al., 2014; Pandey et al., 2020a; Pandey et al., 2020b). This study employed this method to investigate the effect of culture conditions on nutrient removal efficiency and biomass productivity to optimize an ATS system. CO_2_ and light are essential to algal photosynthesis. The nitrogen-to-phosphorous (N:P) ratio has been reported to affect the biochemical composition of algal biomass. Therefore, three independent variables, TIC concentration, N:P ratio, and light intensity were selected to test their independency and interactive effects on nutrient removal, water quality, biomass productivity, and composition in a lab-scale ATS (Figure 1). The experimental ranges of TIC and N:P ratio were selected based on previous studies (Liu and Vyverman, 2015; Li et al., 2019; Zhu et al., 2021). Temperature and light intensity were based on the local climatic conditions (N 50°54′20; E 6°25′4). The annual light intensity of the daily average and maxima were 125 and 359 µmol photons m^−2^ s^−1^, respectively. Thus, the maximum light intensities were 500 and 333 µmol photons m^−2^ s^−1^ in 16 and 24 h, respectively.

## 2 Materials and methods

### 2.1 Culture system

A lab-scale ATS system was designed and constructed using acrylic plates (Figure 1). The 0.1 × 0.52 m flow-way was covered with nylon netting (white, 3.5 × 3.5 mm), serving as a growth substratum. The medium was continuously discharged by a submerged pump at a flow rate of 0.3 L min^−1.^ A tipping bucket distributed the water in a wave-like fashion at an interval of 6–7 s^−1^, to increase the surface contact of the biofilm with air and reduce diffusional resistance (Wilkie and Mulbry, 2002).

### 2.2 Culture conditions

The standard medium was based on the BG11 medium (1 L holds CaCl_2_ ˑ 2 H_2_O, 36 mg; MgSO_4_ ˑ 7 H_2_O, 75 mg; Fe(NH_4_)_3_ (C_6_H_5_O_7_)_2_, 6 mg; EDTA-2 Na, 1 mg; C_6_H_8_O_7_ ˑ H_2_O, 6 mg; H_3_BO_3_, 2.86 mg; MnCl_2_ ˑ 4 H_2_O, 1.81 mg; ZnSO_4_ ˑ 7 H_2_O, 0.22 mg; NaMoO_4_ ˑ 2 H_2_O, 0.39 mg; CuSO_4_ ˑ 5 H_2_O, 0.08 mg; Co(NO_3_)_2_ ˑ 6 H_2_O, 0.05 mg) (Stanier et al., 1971). The standard medium was supplemented with NaNO_3_, K_2_HPO_4,_ and NaHCO_3_ according to the experimental design. 5 L medium was added to the container of each ATS at the beginning of the experiment. Deionized water was regularly added to the system to compensate for evaporation. Each ATS was inoculated with 1 g fresh ATS biomass of a continuous ATS system (wild-type mesocosm, green-house, 3 years), and no pre-selection was performed. The ATS was kept at ambient room temperature and a 16:8 h light:dark cycle. Experiments were conducted in batches for 7 days, after which the biomass was harvested and the dry weight (DW), ash-, C-, N-, and P-contents were recorded. The dissolved oxygen (DO), pH value, total phosphorus (TP), and total nitrogen (TN) in the medium were measured daily.

### 2.3 Box–Behnken design of the experiment

To evaluate the impacts of the three key independent variables—TIC concentration, N:P ratio, and light intensity on the P and N removal efficiencies and biomass productivity—a 3^k^ factorial BBD was applied using the Design-Expert software version 13.0 (STAT-EASE Inc.®, United States ) and R 4.0.5. The three independent variables (symbols: A, B, C) were coded at three levels, namely, low (-1), central (0), and high (+1) (Table 2. TIC concentration was calculated based on the CO_2_ concentration in the atmosphere (Supplementary Table S1). Therefore, 15 treatments were conducted with three replications of the central point for an accurate estimate of pure experimental error (Supplementary Table S2). All treatments were conducted in triplicate (data shown as mean ± standard error). After conducting the experiments, the full quadratic second-order equation with interaction terms was used to model the relationship between dependent and independent variables:

$$y=\beta_{0}+\overset{k}{\underset{i=1}{\sum }}\beta_{i}x_{i}+\overset{k}{\underset{i=1}{\sum }}\beta_{ii}x_{i}^{2}+\overset{k-1}{\underset{i=1}{\sum }}\overset{k}{\underset{j=i+1}{\sum }}\beta_{ij}x_{i}x_{j}+\varepsilon .$$ (1)

**TABLE 2:** Actual and coded levels of the independent variables of the Box–Behnken design (BBD). TIC, total inorganic carbon; N:P, nitrogen-to-phosphorus ratio; TP, total phosphorous.

Independent variables	Symbol	Experimental Values		
		Low (-1)	Central (0)	High (+1)
TIC (mM)	A	1.8	5.4	9
N:P ratio (TP: 10 mg L^−1^)	B	5	10	15
Light Intensity (μE)	C	100	300	500

In Eq. 1, β_0_, β_i_, β_ii_, and β_ij_ are regression coefficients for intercept, linear, quadratic, and interaction coefficients, respectively; x_i_ and x_j_ are coded independent variables; and ε is the residual. The 3D response surface and contour plots were generated to visualize the interactive effects of the independent variables of the responses. The perturbation plots were generated to illustrate the sensitive independent variables. They show one variable over its full range, while fixing all other variables at the midpoint (coded 0). Responsiveness to a variable was indicated by a steep slope or curvature. We applied the numerical optimization function of the Design-Expert software, which uses the desired function of the algorithm, to adjust the growth conditions for maximum nutrient removal efficiency and biomass productivity. At last, the optimized growth conditions were tested experimentally (n = 3) to verify the validity of our model.

### 2.4 Wastewater analysis

A water sample (2 ml) was collected daily from each ATS and filtered (0.45 μm, LCW 916, Hach-Lange®, United States ) before analysis. TP was determined spectrophotometrically (880 nm, SPECORD 200 PLUS, Jena Analytik®, Germany) according to the ammonium molybdate spectrometric method (ISO, 2004). TN was determined spectrophotometrically (220 and 275 nm) following the UV-screening method (Association, 1998). The DO concentration and pH value were measured daily *in situ* using specific sensors and a data-logger (LabQuest 3, Vernier®, United States ).

### 2.5 Biomass analysis

The attached algal biofilm was harvested from the nylon netting at the end of each batch experiment, on day 7. The biofilm was centrifuged at 4,200 *g* at 4°C for 10 min. The supernatant medium was discarded, and the biofilm pellet was stored at −20°C before freeze-drying for DW determination. The suspended biomass was harvested by sampling and filtrating 100 ml of culture medium (1822–047, Whatman®, United States ). The loaded filter was dried to a consistent weight at 70°C for 24 h. The total DW was calculated as the sum of attached and suspended biomass. The total ATS biomass productivity was calculated as follows (Eq. 2):

$$\text{ATS}\;\text{biomass}\;\text{productivity}\;\left(\text{g}\;{\text{m}}^{-2}\;{\text{d}}^{-1}\right)=\;\text{Total}\;\text{DW}\;\left(\text{g}\right)\;/\;\text{ATS}\;\text{area}\;\left({\text{m}}^{2}\right)\;/\;\text{cultivation}\;\text{days}\;\;\left(\text{d}\right).$$ (2)

Ash content was determined by combustion of 100 mg lyophilized biomass in a muffle furnace at 550°C for 2 h (Chen et al., 2015). The C and N contents were determined by elemental analysis (Vario® Elementar, Germany) using 8–10 mg lyophilized biomass. The P-content was determined by inductively coupled plasma—optical emission spectrometry (ICP-OES Ultima 2, HORIBA®, France) in 200 mg lyophilized biomass pretreated with 5 ml HNO_3_ and microwave digestion (MARS6, CEM®, United States ).

## 3 Results and discussion

### 3.1 Water quality and nutrient removal in algal turf scrubber

#### 3.1.1 Dissolved oxygen and pH value

**FIGURE 2:**
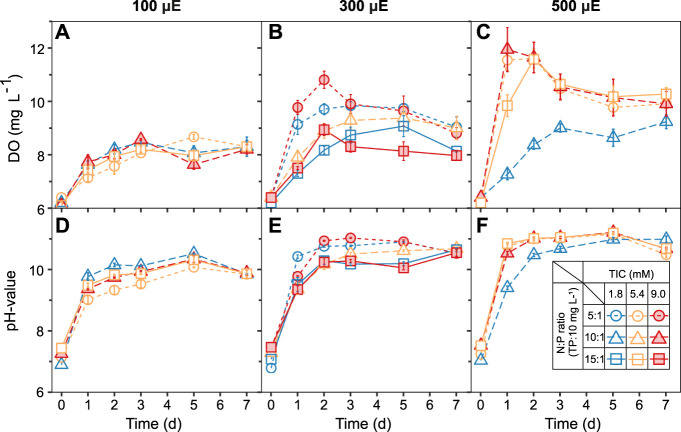
Algal turf scrubber (ATS) biofilms established a stable medium chemistry under all treatments within 3 days. Increasing light intensities to **(A,D)** 100, **(B,E)** 300, and **(C,F)** 500 μmol photons m^−2^ s^−1^ elevated the dissolved oxygen and pH values, respectively. Data are presented as mean ± standard error (SE, n = 3, except the treatment of central point: TIC = 5.4 mM, N:P ratio = 10:1, and light intensity = 300 μmol, n = 9). TIC, total inorganic carbon; N:P, nitrogen-to-phosphorous ratio; TP, total phosphorous.

The effect of selected TIC concentrations, N:P ratios, and light intensities on the ATS biofilm and water quality were monitored *via* DO and pH measurements (Figure 2). Within the first 2 days, the DO concentrations increased from ∼6.5 to 11.6 ± 0.8 mg L^−1^, depending on the light intensity (Figures 2A–C). Between days 3 and 7, the DO concentration leveled off under all light intensities(Figures 2A–C). Under low light intensity (100 μmol photons m^−2^ s^−1^), the DO concentration remained similar for all TIC concentrations and N:P ratios over 7 days (Figure 2A). In comparison, the pH values initially increased in all treatments from ∼7.6 to 9.0 ± 0.1 or 10.9 ± 0.4 over the first day (Figures 2D–F). Between days 2 and 7, all treatments reached stable pH values between 10.5 ± 0.3 to 11.2 ± 0.7, (Figures 2D–F). Similar trends in pH value were reported for outdoor ATS systems, which increased from pH 7.0 to >8.5 within 48 h (Liu et al., 2016).

The lower DO concentrations observed under high N:P ratios (15:1) and medium light (300 µmol) may be due to an increased oxygen consumption during NO_3_^−^ assimilation (Figure 2B) squares (Perez-Garcia et al., 2011). The high DO concentrations under high light intensity (500 μmol) suggest that the ATS biofilm has robust photosynthesis under higher light intensity over a wide range of TIC and N:P ratios (Figure 2C). A simultaneous increase in DO concentration and light intensity during peak times was confirmed previously by Sandefur et al. (2011). The high pH values in our ATS system might be caused by the high CO_2_ uptake, the OH^−^ released from the hydrolysis of HCO_3_^−^, and the strong NO_3_^−^-N consumption by algae biofilm during the growth and photosynthesis (Chi et al., 2011; Perez-Garcia et al., 2011; Xie et al., 2017). We identified a positive correlation between DO concentration and pH value (*R* ^2^ = 0.64), identified by previous studies on ATS systems (Zang et al., 2011; Khan et al., 2019).

### 3.2 Phosphorus and nitrogen removal

**FIGURE 3:**
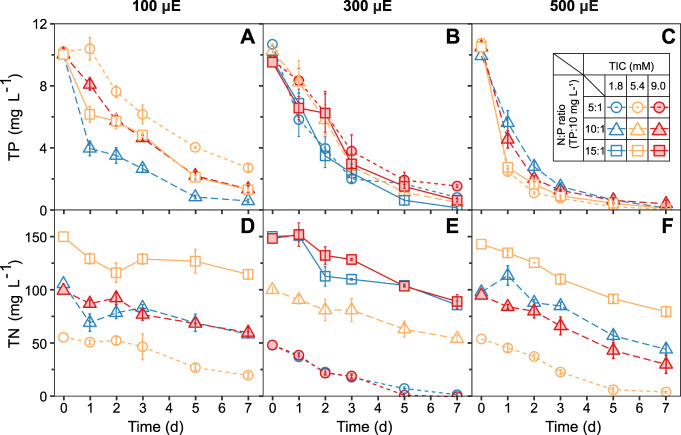
ATS- biofilms showed a continuous nutrient removal from the medium under all treatments over 7 days. Increasing light intensities of **(A,D)** 100, **(B,E)** 300, and **(C,F)** 500 μmol photons m^−2^ s^−1^, respectively, increased the removal of phosphorus and nitrogen. Data are presented as mean ± standard error (SE, n = 3, except the treatment of central point: TIC = 5.4 mM, N:P ratio = 10:1, and light intensity = 300 μmol, n = 9). TIC, total inorganic carbon; N:P, nitrogen-to-phosphorus ratio; TP, total phosphorus; TN, total nitrogen.

The nutrient removal capacity of the ATS system, depending on TIC concentrations, N:P ratios, and light intensities, was monitored daily by measuring the residual TP and TN concentrations in the medium (Figure 3). Within 7 days, the ATS biofilm removed between 7.5 ± 0.2 to 10.4 ± 0.1 mg L^−1^ of TP. The maximum TP removal (99.6 ± 0.4%) was found under TIC 5.4 mM, N:P ratio 5:1, and high light intensity of 500 μE (Figure 3C circles). Approximately 80% of TP was removed within 24 h (Figure 3C). As a consequence, the lowest TP removal (73.4 ± 2.3%) was found under low light (Figure 3A). Likewise, within 7 days, the ATS biofilm removed between 35.2 ± 4.5 and 64.7 ± 3.8 mg L^−1^ TN (Figures 3D–F). The maximum TN removal (100%) occurred under TIC 9.0 mM, N:P ratio 5:1, and light intensity of 300 μmol photons m^−2^ s^−1^ (Figure 3E) circles.

In this study, the maximum TP removal rate (8.25 mg L^−1^ d^−1^) was two-fold higher than previously reported in an outdoor ATS system (3.9 mg L^−1^ d^−1^) (Liu et al., 2016) and six-fold higher than an indoor algal biofilm system (1.3 mg L^−1^ d^−1^) (Shi et al., 2007). It is known that algae have various mechanisms to assimilate, absorb, and precipitate P out of the medium (Su et al., 2011; Xu et al., 2014). At optimal pH values between 9 and 11, the P-adsorption to the algal cell wall can occur within minutes (Nurdogan and Oswald, 1995; Sañudo-Wilhelmy et al., 2004). The high pH values (pH > 9 after 24 h) and high P removal rate in our ATS system confirmed that the P precipitation and adsorption were high in our biofilm. Likewise, our maximum TN removal rate (19.1 mg L^−1^ d^−1^) was six-fold higher than the previously reported 3.1 mg L^−1^ d^−1^ for the algal biofilm system (Shi et al., 2007). In contrast to P, the N-uptake in algae is an energy-dependent assimilation process (Perez-Garcia et al., 2011). In a highly light-dependent, stepwise reduction process, eukaryotic algae reduce NO_3_^−^ to NO_2_^−^ and NH_4_^+^ in their cytosol and chloroplasts, respectively (Sanz-Luque et al., 2015; Su, 2020). Therefore, we found the highest nitrogen removal rates in the ATS under high light conditions, Figure 3C.

### 3.3 Culture conditions for improved nutrient removal

#### 3.3.1 Statistical analysis

The relationship between the three independent and six dependent variables (responses) was analyzed using RSM. The two-factor interaction and the quadratic model were used for data fitting. The final model equations, cleared of insignificant variables and interactions, and the analysis of variance results for the responses, are shown in Table 3.

**TABLE 3:** Analysis of variance for the applied response surface model. TP, total phosphorus; TN, total nitrogen; A, total inorganic carbon (TIC); B, nitrogen-to-phosphorus ratio (N:P ratio); C, light intensity; *R* ^2^, determination coefficient; a.*R* ^2^, adjusted *R* ^2^; a.P., adequate precision; SD, standard deviation; CV, coefficient of variation.

Responses	Modified Equations with Significant Terms	Probability	*R* ^2^	a.*R* ^2^	a.P	SD	CV (%)	Sum of Squares		Probability for Lack of Fit
								Pure error	Lack of fit	
TP removal (%)	94.54–2.96A+ 3.56B+ 6.56C - 3.52BC - 3.67B^2^	<0.01	0.83	0.74	11.5	3.67	3.96	23.00	98.28	0.52
TN removal (%)	45.78–1.15A-25.22B+ 10.93C–4.61AC + 9.89 A^2^ + 14.05B^2^ -3.92C^2^	<0.01	0.98	0.96	22.9	4.55	8.06	21.03	123.89	0.32
Productivity (g m^−2^ d^−1^)	6.77 + 0.65A+ 0.3B+ 2.62C - 0.49B^2^ + 0.7C^2^	<0.01	0.98	0.97	30.0	0.35	5.12	0.14	0.98	0.37
P content (%)	1.78 + 0.05A+ 0.02B- 0.11C - 0.26AB+ 0.07BC - 0.13A^2^ - 0.14B^2^ - 0.14C^2^	0.01	0.93	0.84	9.3	0.08	5.49	0.01	0.03	0.61
N content (%)	7.14–0.08A+ 0.58B- 0.51C + 0.61BC - 0.68B^2^ + 0.38C^2^	<0.01	0.94	0.90	16.7	0.26	3.73	0.16	0.38	0.65
Ash content (%)	8.18–0.62A+ 0.05B- 1.14C - 0.31AB- 0.44AC	<0.01	0.93	0.89	15.9	0.35	4.28	0.14	0.96	0.38

For all responses, the low probability values (≤0.01) revealed that the generated models were significant. Experimental results were well aligned with the generated models as confirmed by analyzing predicted against measured values (Supplementary Figure S1). For all six responses, most of the points were within the 95% confidence interval region. Adequate precision was measured by the signal-to-noise ratio and a value >four was desirable for good discrimination. All generated models met this requirement. Meanwhile, low variation coefficients (3.7–8.1%) indicated a high precision and experimental reliability for all models. The *F*-test of sum of squares to lack of fit confirmed the adequacy of our quadratic model. A *p*-value of lack of fit greater than 0.05 (>0.32) implied that the *F*-statistic was insignificant for all the models. A detailed analysis of the response models is presented in the following sections.

### 3.4 Phosphorus removal

**FIGURE 4:**
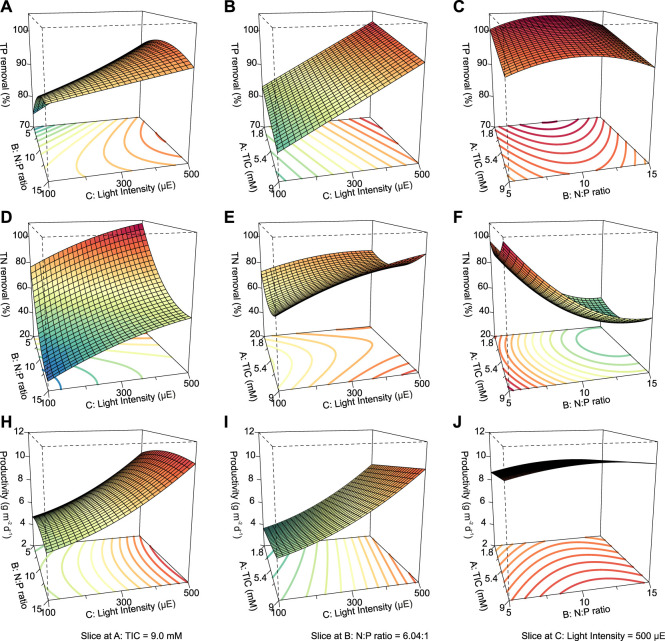
. 3D response surface and contour plots visualizing the interactive effects between the TIC concentration, N:P ratio, and light intensity for ATS performance. TP removal, TN removal, and biomass productivity are shown as a function of N:P ratio vs. light intensity at a fixed TIC concentration of 9.0 mM **(A,D,H)**; TIC concentration vs. light intensity at a fixed N:P ratio of 6.04:1 **(B,E,I)**; TIC concentration vs. N:P ratio at a fixed light intensity of 500 μmol photons m^−2^ s^−1^ **(C,F,J)**, respectively. TIC, total inorganic carbon; N:P, nitrogen-to-phosphorus ratio; TP, total phosphorus; TN, total nitrogen.

**FIGURE 5:**
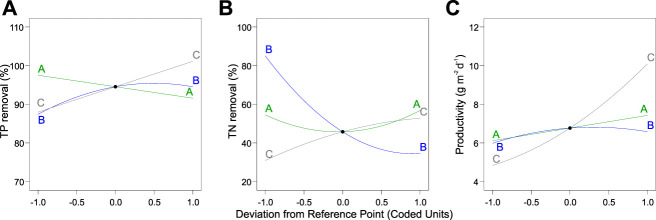
Perturbation plot of the dependent variables **(A)** total phosphorus and **(B)** total nitrogen as well as **(C)** biomass productivity. Legend: A, total inorganic carbon; B, nitrogen-to-phosphorus ratio; C, light intensity.

The independence and interdependency of the different variables were analyzed to determine the relationships between the TP removal and cultivation conditions. 3D response surfaces and contour plots obtained using the quadratic model were generated (Figure 4). One variable was kept at an optimal level, and two variables were allowed to vary within the experimental range (Figure 4). The curvatures revealed that there was a strong interactive effect between the N:P ratio and light intensity (Figure 4A). The N:P ratio showed an optimum of the TP removal efficiency, while the efficiency decreased at larger and smaller ratios (Figures 4A, C). The individual effects of three independent variables on the TP removal are visualized via the perturbation plot (Figure 5A). The TP removal was sensitive to all three independent variables (Figure 5A). Moreover, the light intensity (term C) had the highest coefficient in the modified model equation (Table 2). It was the most significant variable for TP removal under all tested light intensities. This is contrary to single-cell cultures, where similar light intensities can be harmful and decrease the TP removal (Al Ketife et al., 2017).

### 3.5 Nitrogen removal

Likewise, the relationships between the TN removal efficiency and cultivation conditions were analyzed. 3D response surface and contour and perturbation plots obtained by the quadratic model of Table 3 are presented in Figures 4D–F and Figure 5, respectively. The interactive effect of TIC concentration and light intensity is displayed in Figure 4E. The TN removal efficiency was more sensitive to the N:P ratio and light intensity than TIC concentration (Figure 5B), as confirmed by the linear model coefficients. High initial TN concentration may contribute to lower removal efficiency. This is consistent with previous studies showing that TN removal efficiency in algae dropped under high N:P ratios (Xin et al., 2010; Liu and Vyverman, 2015). Moreover, the significant positive effect of light intensity on TN removal efficiency confirmed that the NO_3_^−^ -N assimilation in algae is an energetically expensive process (Perez-Garcia et al., 2011).

### 3.6 Biomass productivity

The third performance indicator for the ATS effectivity was biomass productivity. Overall, the productivity of ATS was 4.14–11.23 g m^−2^ d^−1^ under all the treatments, Supplementary Table S2. Our results are in line with other studies, despite the wide range of ATS productivities (2–49 g m^−2^ d^−1^) due to the different cultivation and nutrient conditions (Walter et al., 2008; Liu et al., 2016; Marella et al., 2019).

Parameters of the quadratic model to biomass productivity are presented in Table 3. The three-dimensional response surface and contour plots are shown in Figures 4H–J and Figure 5C, respectively. The biomass productivity was sensitive to all three independent variables (Figure 5C). Again, light intensity had a strong positive effect and showed the highest coefficient in the model equation (Table 3). This aligns with previous studies demonstrating the increased light resilience of algal biofilm compared to single-cell cultures (Al Ketife et al., 2017). Thus, photosynthetic bacteria and algae stratify within a biofilm matrix according to the light availability and their preference (Thapa et al., 2017). In addition, the TIC concentration had a significant positive effect on ATS biomass productivity. Although, to the best of our knowledge, there are no publications reporting on the effect of TIC concentration on ATS biomass productivity, it has been shown that bicarbonate can promote higher biomass productivity (Su, 2020; Zhu et al., 2021).

#### 3.6.1 Biomass P, N, and ash contents

For a subsequent valorization of the nutrient-rich biomass, the P-, N-, and ash contents were quantified under different growth conditions (Supplementary Figures S2A–J). The P-content ranged from 1.1 ± 0.1 to 1.9 ± 0.2% DW (Supplementary Figures S2A–C). Similar *p*-values of 0.9–3.2% DW were reported for ATS biomass using municipal WW (P: 3.7–4.4 mg L^−1^). In addition, we identified a significant interactive effect for the P-content between TIC concentration and the N:P ratio (Supplementary Figure S2C).

The N-content reached 7.8 ± 0.2% DW (Supplementary Figures S2D–F). At this level, we identified a significant interactive effect on the N-content by light intensity and N:P ratio in the medium (Supplementary Figure S2D). In particular, the N-content reached its maximum at a medium N:P ratio of 10:1, while a higher N:P ratio (15:1) caused a reduced N-content of the biomass (Supplementary Figure S2D).

The ash content of ATS biomasses ranged from 6.4 ± 0.2 to 9.5 ± 0.4% DW (Supplementary Figures S2H–J). These values are six- to 10-fold lower than those previously reported for ATS biomass grown in agricultural drainage or reservoir water (Kangas and Mulbry, 2014; Chen et al., 2015). Based on microscopic observations, it is suggested the low ash content was due to the low number of diatomaceous sediments and suspended solids, the major contributors to the ash. Moreover, we found an inverse correlation (*R* ^2^ = −0.73) between biomass productivity and ash content. Increased growth at high TIC concentrations and light intensity decreased the final ash content of the biomass (Supplementary Figure S2I).

### 3.7 Process optimization

Using the BBD–RSM, the optimal cultivation conditions (TIC 5 mM, N:P ratio 6.04, light intensity 500 μE) were identified to simultaneously maximize the TP and TN removal, as well as the biomass productivity. Accuracy of the optimal conditions was confirmed by experimental data within the prediction interval (PI) and in proximity to the predicted values (Table 4. It should be noted that the optima of TIC concentration and light intensity were both located at the very limit of the chosen range. However, under these optimal conditions, the predicted nutrient removal was very close to 100% (Table 4). Considering that light was the most important variable in ATS performance, we experimented with the higher light intensity of 1,000 µmol photons m^−2^ s^−1^. We then set the other variables at their predicted optimal values. We found no significant differences (*p* < 0.5) between the TP and TN removal or biomass productivity at the 500 and 1,000 µmol photons m^−2^ s^−1^, respectively (Supplementary Table S3). This indicates that 500 µmol photons m^−2^ s^−1^ light intensity is close to the saturation level for nutrient removal in our ATS system.

**TABLE 4:** Validation results under optimized growth conditions of TIC (9 mM), N:P ratio (6.04, TP 10 mg L^−1^), and light intensity (500 μmol photons m^−2^ s^−1^). Data are presented as mean ± standard error (SE, n = 3). TP, total phosphorus; TN, total nitrogen; PI, prediction interval.

Responses	Experimental (mean ± SE)	95% PI (low)	Predicted	95% PI (high)	Error (%)
TP removal (%)	97.25 ± 0.81	82.27	95.85	109.43	1.44
TN removal (%)	91.25 ± 0.44	84.93	100.0	115.07	8.75
Productivity (g m^−2^ d^−1^)	10.58 ± 0.28	9.06	10.22	11.38	3.40

## 4 Conclusion

The ATS is a promising algal-based WW treatment technology. It can achieve high nutrient removal in a short time. This study successfully demonstrated the RSM-based optimization of both nutrient uptake and biomass productivity in a lab-scale ATS. Up to 80% of phosphorus was removed within 24 h. We were able to show the correlation of independent variables such as TIC, N:P, and light with nutrient removal and biomass production. Ongoing studies utilize these findings to optimize nutrient removal at a production scale in ATS systems at WW treatment facilities and using other WW types.

## Data Availability

The original contributions presented in the study are included in the article/supplementary material. Further inquiries can be directed to the corresponding author.
