# Supplementary material for: Optimizing nutrient removal and biomass production of the Algal Turf Scrubber (ATS) under variable cultivation conditions by using Response Surface Methodology
Source: Front Bioeng Biotechnol. 2022 Sep 6;10:962719. doi: 10.3389/fbioe.2022.962719 (PMC9486005; doi:10.3389/fbioe.2022.962719)
Supplement: Supplementary file 1 [file DataSheet1.pdf]

Supplementary Material

1 Supplementary Figures and Tables

1.1 Supplementary Figures

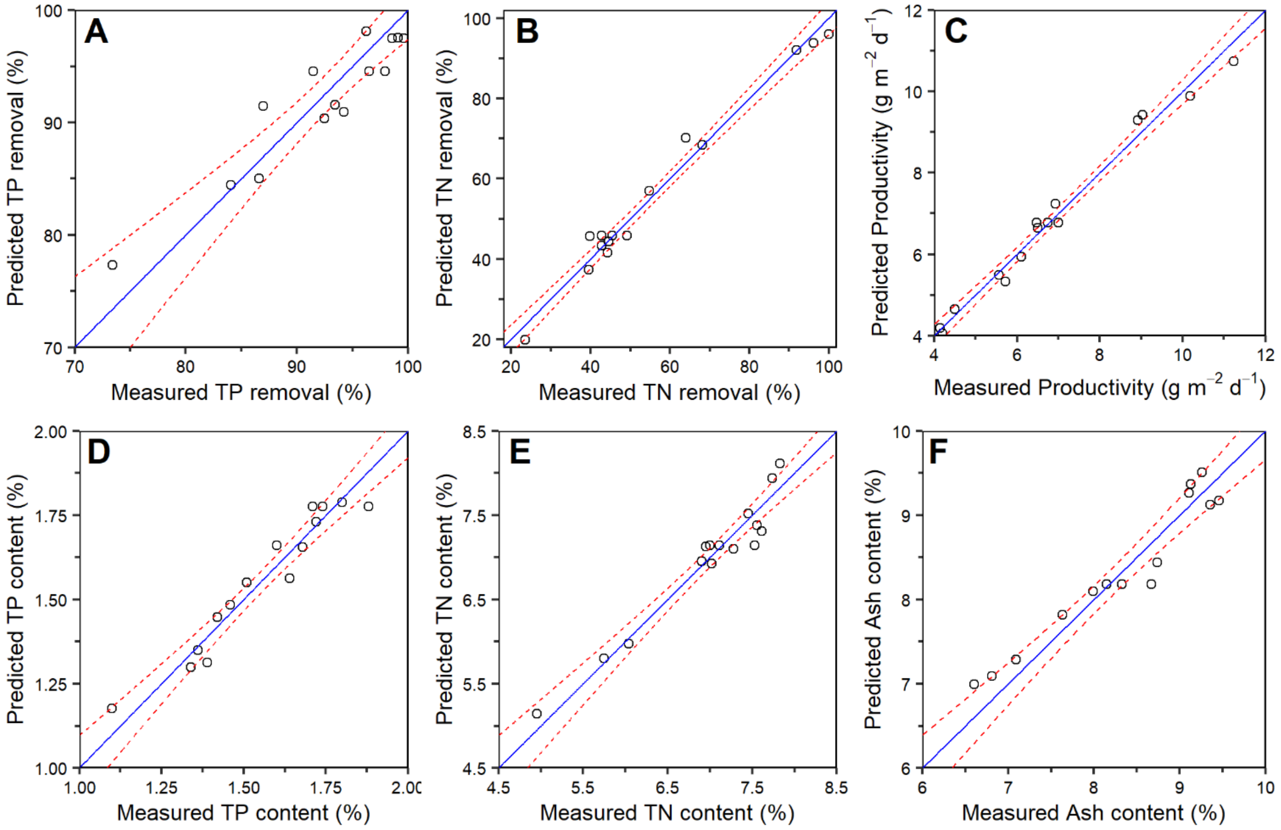

Figure S 1. Recovery rates of predicted and measured dependent variables. Tested were (A) Phosphorus and (B) Nitrogen removal from the medium. And the biomass (C) productivity, and its (D) phosphorus, (E) nitrogen, and (F) ash content, respectively. (—) full recovery of 100%, (- - -) Regression line with 95% confidence interval.

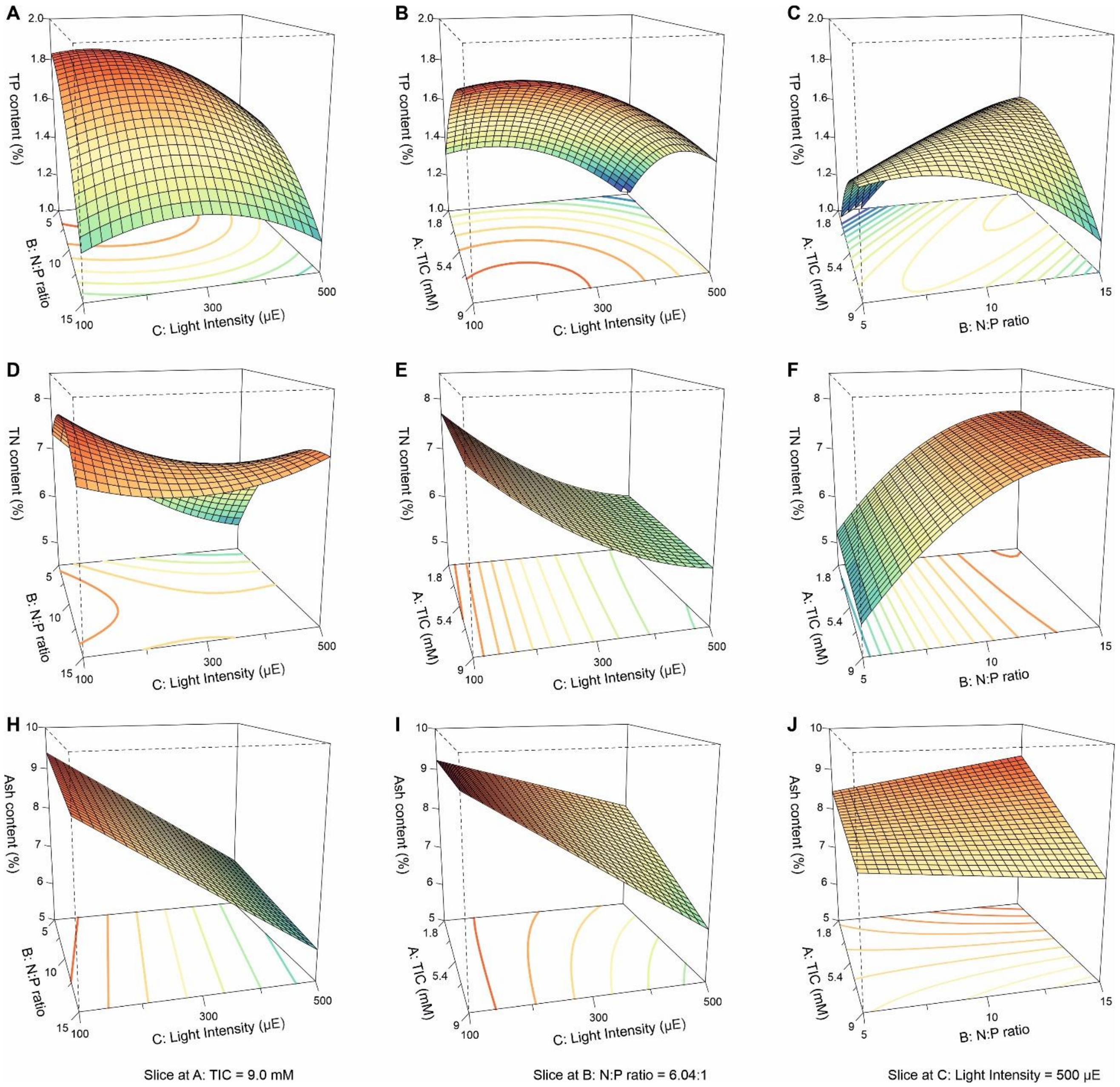

Figure S 2. 3D response surface and contour plots visualizing the interactive effects between TIC concentration, N:P ratio and light intensity for ATS biofilm composition. TP, TN and ash content are shown as a function of N:P ratio vs. light intensity at fixed TIC concentration of 9.0 mM (A, D, H); TIC concentration vs. light intensity at fixed N:P ratio of 6.04:1 (B, E, I); TIC concentration vs. N:P ratio at fixed light intensity of 500 μE (C, F, J), respectively. TIC, Total inorganic carbon; N:P, Nitrogen: Phosphorus ratio; TP, Total phosphorus; TN, Total nitrogen.

1.2 Supplementary Tables

Table S 1. Transfer of atmospheric CO_2 into the culture medium by bubbling.

CO_2 concentration C_{CO2} (ppm)	CO_2 volume by bubbling V_{CO2} (mL) $= C \div 10^6 \times 50$ mL min$^{-1} \times 10080$ min	CO_2 amount N_C (mmol) $= V_{CO2} \div 22.4$ L mol^{-1}	CO_2 molarity c_{CO2} (mM) $= N_C \div 5$ L
407.0	205.13	9.16	1.8
1203.5	606.56	27.08	5.4
2000.0	1008.00	45.00	9.0

Table S 2. BBD experimental data of the independent variables (coded factors) and of the dependent variables (responses). Data are presented in mean ± SE (n = 3). TP, Total phosphorus; TN, Total nitrogen.

Run	Coded factors			Responses					
	A	B	C	TP removal (%)	TN removal (%)	Productivity (g m^{-2} d^{-1})	TP content (%)	TN content (%)	Ash content (%)
1	-1	-1	0	92.5 ± 0.88	96.2 ± 2.38	5.72 ± 0.39	1.09 ± 0.04	6.04 ± 0.21	8.74 ± 0.11
2	1	-1	0	84.0 ± 0.84	100 ± 0	6.49 ± 0.51	1.79 ± 0.01	5.74 ± 0.08	7.63 ± 0.11
3	-1	1	0	98.5 ± 0.81	42.8 ± 2.52	6.09 ± 0.39	1.71 ± 0.13	6.94 ± 0.09	9.45 ± 0.35
4	1	1	0	93.4 ± 1.22	39.8 ± 4.29	6.93 ± 0.48	1.38 ± 0.16	6.90 ± 0.29	7.09 ± 0.85
5	-1	0	-1	94.2 ± 1.08	44.5 ± 2.15	4.14 ± 0.36	1.63 ± 0.05	7.82 ± 0.18	9.25 ± 0.17
6	1	0	-1	86.6 ± 2.24	39.5 ± 4.90	5.56 ± 0.61	1.60 ± 0.03	7.74 ± 0.19	9.36 ± 0.10
7	-1	0	1	98.7 ± 0.28	54.7 ± 5.36	9.02 ± 0.72	1.35 ± 0.08	7.27 ± 0.00	7.98 ± 0.20
8	1	0	1	96.2 ± 2.27	68.1 ± 8.88	11.2 ± 0.46	1.42 ± 0.13	7.01 ± 0.07	6.35 ± 0.19
9	0	-1	-1	73.4 ± 2.34	64.0 ± 4.38	4.18 ± 0.34	1.68 ± 0.25	7.56 ± 0.32	9.11 ± 0.30
10	0	1	-1	86.9 ± 1.16	23.5 ± 3.01	4.49 ± 0.24	1.50 ± 0.10	7.61 ± 0.07	9.13 ± 0.47
11	0	-1	1	99.6 ± 0.37	91.9 ± 1.41	8.92 ± 0.28	1.34 ± 0.08	4.94 ± 0.05	6.59 ± 0.26
12	0	1	1	99.1 ± 0.88	44.2 ± 3.47	10.1 ± 0.48	1.45 ± 0.21	7.44 ± 0.05	6.81 ± 0.30
13	0	0	0	97.9 ± 0.85	49.2 ± 6.46	6.46 ± 0.56	1.71 ± 0.22	6.99 ± 0.18	8.33 ± 1.13
14	0	0	0	96.4 ± 0.73	45.3 ± 2.30	6.73 ± 0.13	1.73 ± 0.12	7.52 ± 0.02	8.15 ± 0.21
15	0	0	0	91.4 ± 2.40	42.7 ± 4.17	6.99 ± 0.82	1.87 ± 0.15	7.10 ± 0.06	8.67 ± 0.09

Table S 3. Results under high light (1000 μE) and optimized growth conditions of TIC (9 mM) and N:P ratio (6.04, TP 10 mg L^{-1}). Data are presented as mean ± standard error (SE, n = 3). TP, Total phosphorus; TN, Total nitrogen.

Responses	Experimental (mean ± SE)	Independent t-test p^*
TP removal (%)	96.05 ± 1.09	0.496
TN removal (%)	92.75 ± 0.41	0.067
Productivity (g m^{-2} d^{-1})	10.96 ± 0.35	0.445
